# Structure and Expression of Large (+)RNA Genomes of Viruses of Higher Eukaryotes

**DOI:** 10.1134/S0006297921030020

**Published:** 2020-12-30

**Authors:** Alexey A. Agranovsky

**Affiliations:** Faculty of Biology, Lomonosov Moscow State University, 119234 Moscow, Russia

**Keywords:** viral positive-sense RNA genomes, nidoviruses, SARS-CoV, closteroviruses, evolution, gene expression

## Abstract

Viral positive-sense RNA genomes evolve rapidly due to the high mutation rates during replication and RNA recombination, which allowing the viruses to acquire and modify genes for their adaptation. The size of RNA genome is limited by several factors, including low fidelity of RNA polymerases and packaging constraints. However, the 12-kb size limit is exceeded in the two groups of eukaryotic (+)RNA viruses – animal nidoviruses and plant closteroviruses. These virus groups have several traits in common. Their genomes contain 5′-proximal genes that are expressed via ribosomal frameshifting and encode one or two papain-like protease domains, membrane-binding domain(s), methyltransferase, RNA helicase, and RNA polymerase. In addition, some nidoviruses (i.e., coronaviruses) contain replication-associated domains, such as proofreading exonuclease, putative primase, nucleotidyltransferase, and endonuclease. In both nidoviruses and closteroviruses, the 3′-terminal part of the genome contains genes for structural and accessory proteins expressed via a nested set of coterminal subgenomic RNAs. Coronaviruses and closteroviruses have evolved to form flexuous helically symmetrical nucleocapsids as a mean to resolve packaging constraints. Since phylogenetic reconstructions of the RNA polymerase domains indicate only a marginal relationship between the nidoviruses and closteroviruses, their similar properties likely have evolved convergently, along with the increase in the genome size.

## INTRODUCTION

Rapid evolution of viral RNA genomes, which is due to the high mutation rates and gene shuffling during RNA replication, leads to the acquisition and modification of genes involved in virus adaptation. The majority of RNA viruses have compact genomes 4 to 12 kb in size [1]. The size of an RNA genome is limited by a number of factors, the major one being the low replication fidelity. Viral RNA-dependent RNA polymerases introduce approximately 10^–4^ errors per nucleotide, which is several orders of magnitude more than the error rate of DNA polymerases [2, 3]. It is believed that accumulation of unfavorable mutations during replication of large RNA genomes may define a threshold for the reproduction of viable virus variants [4]. In addition, the size of viral RNA influences its stability and packaging [5].

In the course of evolution, RNA genomes of animal nidoviruses (families *Arteriviridae*, *Coronaviridae*, *Roniviridae*, and *Mesoniviridae*; order Nidovirales) and plant closteroviruses (fam. *Closteroviridae*) have exceeded the 12-kb limit. Coronaviruses have the largest undivided RNA genomes among all known (+)RNA viruses (up to 41 kb [6]). The size of closteroviral genomes ranges from 14.5 to 19 kb [7, 8]. This review is focused on the structure, encapsidation, replication, and expression of large RNA genomes. Comparison of nidoviruses and closteroviruses reveals paradoxical similarities between these evolutionary distant groups of animal and plant viruses.

## CORONAVIRUSES

Members of the order Nidovirales – arteriviruses, coronaviruses, mesoniviruses, and roniviruses – show considerable variation in the genome size and structure. Comparison of amino acid sequences of the most conserved virus enzyme, RNA polymerase, indicates that nidoviruses form a compact phylogenetic cluster [9] in the picorna-like phylum [10]. This review is limited to the discussions of *Coronaviridae* viruses only as the most studied and epidemiologically important family of the Nidovirales order.

**Fig. 1.**
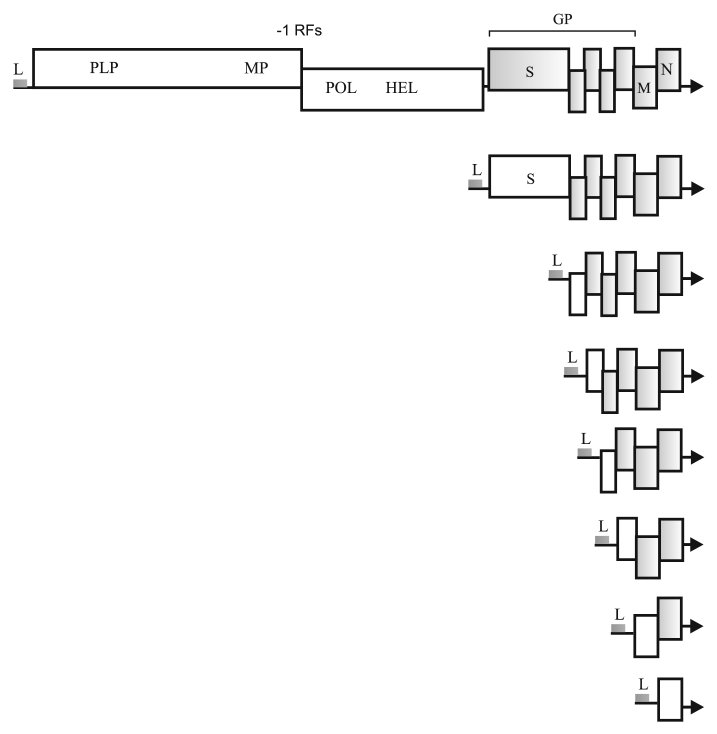
Genomic RNA and sgRNAs of SARS-CoVs: L, leader sequence; -1 RFs, -1 ribosomal frameshifting signal. Encoded proteins and protein domains: PLP, papain-like cysteine protease; MP, (main) serine protease; POL, RNA polymerase; HEL, RNA helicase; S, spike glycoprotein; GP, accessory proteins and outer membrane glycoprotein; M, matrix protein; N, nucleocapsid protein. Genes unavailable for translation in each type of mRNA are shown as shaded boxes. Arrows indicate 3′-ends of RNAs. Drawn approximately to scale.

**Genome structure and expression.** Coronaviruses have helically symmetrical nucleocapsids surrounded by the lipoprotein membrane containing the spike (S) glycoprotein and other viral glycoproteins [9]. The genomic RNA has the 5′-terminal cap and the 3′-terminal poly(A) tract. ORFs 1a and 1b, coding for the replicase components, occupy the 5′-proximal portion of the genome (Fig. 1). Translation of these genes via the -1 ribosomal frameshifting yields 1a and 1ab polyproteins (pps) of ~4,000 and ~7,000 aa, respectively, at an approximate ratio of 4 : 1. The processing of these polyproteins by the viral proteases yields 16 nonstructural proteins (nsps). The genes for the structural proteins – membrane glycoproteins, matrix protein (M), and nucleocapsid protein (N) – map to the 3′-terminal genome portion and are expressed via a nested set of 3′-coterminal subgenomic RNAs (sgRNAs) (Fig. 1). The genomic RNA and sgRNAs have identical 5′-terminal leader sequences (L) of 60-90 nt.

**Fig. 2.**
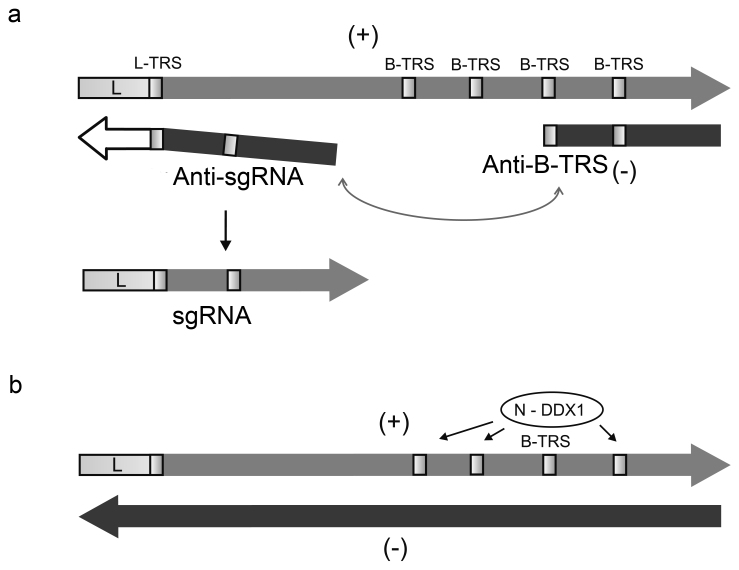
Transcription and replication of coronaviral RNA. a) Discontinuous transcription. (–)RNA synthesis on the genomic (+)RNA may stop on the transcription regulatory signal (B-TRS, light box), with the following transition of the nascent (–)strand to the 5′-leader, annealing of B-TRS to L-TRS, and copying of the leader sequence. Anti-sgRNAs serve as templates to produce sgRNAs. b) Continuous replication. Free viral nucleocapsid protein N^0^ and cell helicase DDX1 attach to B-TRSs, allowing replicase to ignore the stop signals and to synthesize the full-length (–)RNA, which serves as a template for producing progeny (+)RNAs. The RNA 3′-ends are shown by arrowheads. Coding and noncoding sequences are drawn not to scale.

The 3′-terminal genes for the accessory and structural proteins are expressed via sgRNAs. In each sgRNA, only the most 5′-terminal gene is available for the ribosomes (Fig. 2). The sgRNAs are synthesized by the unique discontinuous transcription mechanism [11, 12]. Upstream of each viral ORF (except ORF 1b) in the genomic RNA, there is a conserved transcriptional regulatory signal (TRS) 6 to 8 nt in length: L-TRS (leader TRS) or B-TRS (body TRS) (Fig. 2). The synthesis of antigenomic (–)RNA might stall at the B-TRS in the template genomic (Fig. 2). Then the (–)RNA strand “jumps” to the 5′-terminal leader of the template strand or, more likely, the leader and the (–)RNA are brought in proximity to each other due to the looping of the template (+)RNA. This is followed by annealing of the anti-B-TRS and L-TRS and completion of the (–)RNA strand on the (+)L template (Fig. 2a). The resulting anti-sgRNAs, containing common anti-L sequence, serve as templates for the generation of sgRNAs. Phosphorylated free nucleocapsid protein N^0^ binds to the B-TRS and recruits cell helicase DDX1. These interactions at the late stage of the infection cycle allow the replicative complex to bypass the TRSs in the template genomic RNA and to synthesize the full-length (–)RNA serving as a template for the generation of progeny (+)strands (Fig. 2b) [13, 14].

**Fig. 3.**
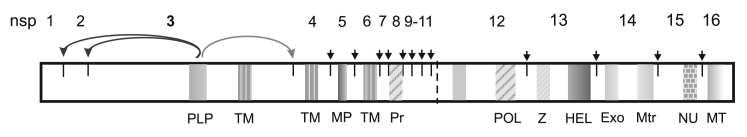
Structure of the SARS-CoV-1 replicative pp1ab. Vertical dotted line indicates the boundary between 1a and 1b in the pp1ab protein. The domains for the nonstructural proteins nsp1-16 are shown as filled boxes. Designations: PLP, papain-like protease (*in cis* cleavage sites are indicated by curved arrows); MP, main chymotrypsin-like protease (*in trans* cleavage sites are shown by arrowheads); TM, transmembrane domain; Pr, putative primase; POL, RNA polymerase; Z, zinc-binding domain; HEL, RNA helicase; Exo, 3′-5′ exonuclease; Mtr, N^7^-guanine methyltransferase; NU, nidoviral uridylate-specific endoribonuclease; MT, 2′-O-ribose methyltransferase.

**Replication-associated proteins.** The replicative complex of coronaviruses consists of 16 nonstructural proteins produced by the processing of pp1a and pp1ab by the viral proteases (Fig. 3). Closely related coronaviruses may have one or two PLP domains [9]. In mouse hepatitis virus (MHV), the autocatalytic release of nsp1, nsp2, nsp3, and nsp4 is carried out by two PLP domains, whereas in SARS-CoV-1, SARS-CoV-2, MERS (Middle East respiratory syndrome)-CoV, and infectious bronchitis virus (IBV), these cleavages are mediated by a single PLP (Figs. 1and 3). It is possible that the PLP domains have originated by duplication and then diverged in the course of coronavirus evolution. The majority of the cleaved bonds in the coronaviral polyproteins are hydrolyzed *in trans* by the main chymotrypsin-like proteinase (MP) [9]. Specific functions of coronaviral nsp1 and nsp2 are apparently associated with the degradation of cell mRNAs and inhibition of their translation [15, 16] and with the maturation of viral particles [17]. Point mutations blocking the nsp1/nsp2 cleavage site and even in-frame deletions of nsp1 and nsp2 have only a limited effect on the MHV replication in the cell culture [18].

**Fig. 4.**
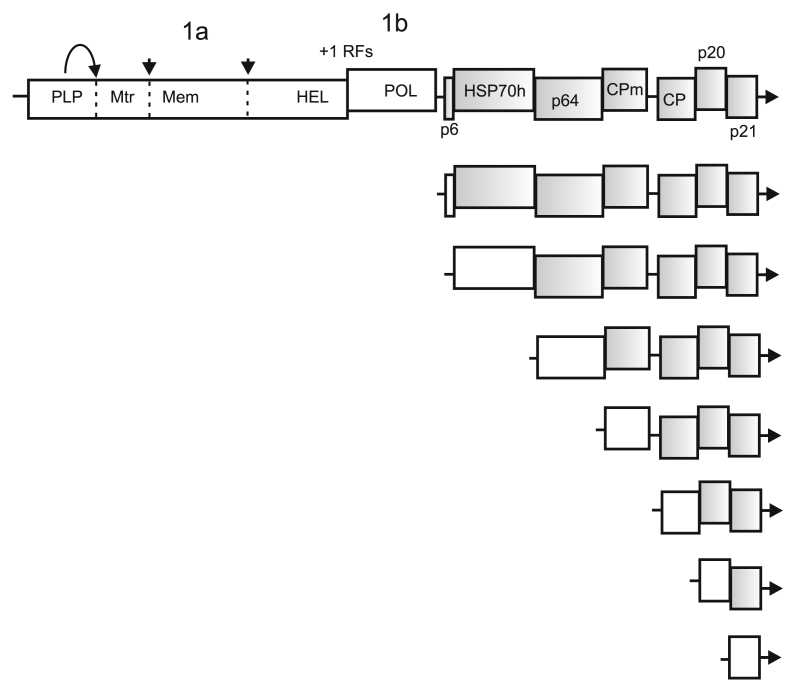
Genomic RNA and sgRNAs of beet yellows closterovirus (BYV). Genes unavailable for translation in each type of mRNA are shown as shaded boxes; +1 RFs, +1 ribosomal frameshifting signal for the translation of ORFs 1a and 1b; arrows and dotted lines indicate cleavage sites in pp1a; Mem, membrane-binding domain; p6, small hydrophobic protein; HSP70h, homolog of HSP70 family heat-shock proteins; p64, 64-kDa protein; CPm, minor capsid protein; CP, major capsid protein.

The multifunctional nsp3 contains PLP and transmembrane (TM) domains [19]. The TM proteins nsp3, nsp4, and nsp6 induce reorganization of cell membranes and formation of replication compartments [20-22]. The nsp5 protease (MP) performs the majority of cleavages in the 1a and 1ab polyproteins (Fig. 4). Mature nsp5 is a part of the replicative complex. Small proteins nsp7-11 are involved in the RNA synthesis; nsp7 and nsp8 form a cylindrical heterooctameric complex, in which positively charged amino acid residues are exposed to the central lumen. A unique feature of coronaviruses, first demonstrated for SARS-CoV-1, is the presence of accessory RNA polymerase nsp8 (hypothetical primase, Pr; Fig. 3) [23]; nsp8 uses the consensus RNA sequence 5′-(G/U)CC as a template and synthesizes short complementary RNAs (up to 6 nt). In addition to poor processivity, nsp8 has the lowest fidelity among all known RNA polymerases (one misincorporation per 10 nt). It was suggested that nsp8 may act as a primase by synthesizing short RNA primers, or as a cofactor that increases the processivity of the RNA polymerase complex [23, 24].

The core RNA-synthesizing enzyme of coronaviruses nsp12 contains nucleotidyltransferase and RNA polymerase domains [24]; nsp13 contains the zinc-binding and helicase domains (Fig. 4).

The nsp14 protein includes the N-terminal 3′-5′ exonuclease domain (Exo) and the C-terminal N^7^-guanine methyltransferase (Mtr) domain (Fig. 3). The activity of these domains, first predicted by computer methods, was later confirmed experimentally [25-27]. Cell exonucleases of the DEDD family, which are related to the coronaviral Exo, participate in the proofreading, repair, and recombination of nucleic acids. The nsp14 protein acts as a proofreading enzyme in the replication of coronaviral RNA and increases the fidelity of RNA copying; this function is unique for large nidoviruses [25, 26].

The nsp15 contains uridylate-specific endoribonuclease (NU) domain (Fig. 3) [28], which is necessary for the replication and transcription and plays a role of interferon antagonist. The nsp16 contains 2′-O-ribose methyltransferase (MT) domain that methylates residue adjacent to the 5′-cap (m^7^GpppAm) in viral mRNA [29]. 2′-O-methylation allows the cell to recognize foreign mRNAs with the help of interferon-induced IFIT proteins; it is possible that 2′-O-methylation helps coronaviruses to evade this restriction mechanism [30].

**Cytology of replication.** After entering the cell, the coronavirus nucleocapsid is transported to the endosomes, where genomic (+)RNA is released from the N protein. Free genomic (+)RNA enters the cytoplasm and is directed to the ribosomes [31] for the synthesis of pp1a and pp1b, which are then cleaved by viral proteases to yield replication-associated proteins. Hydrophobic proteins nsp3, nsp4, and nsp6 bind to the Golgi and ER membranes and, apparently, with the aid of cell protein partners, generate replication compartments, such as double-membrane vesicles (DMVs) and multivesicular complexes (MVCs) [20, 21]. Coronavirus nonstructural proteins and RNA bound to the DMV and MVC membranes form the “viral factories” that serve as sites for the synthesis of coronaviral antigenomic RNAs, progeny genomic RNAs, and sgRNAs [22]. Genomic and subgenomic RNAs migrate from the viral factories to the cytoplasm, where they are used as templates for translation and replication. The replication compartments induced by MHV and SARS CoV-2 have channels formed by nsp3 that open to the cytoplasm and serve for the import of substrate ribonucleoside triphosphates (rNTPs) and export of viral mRNAs [32].

## CLOSTEROVIRUSES

The *Closteroviridae* family includes about 40 plant (+)RNA viruses [33, 34] characterized by several traits, such as (i) semi-persistent mode of insect transmission (virus inhabits its vector for a few hours); (ii) unique structure of filamentous particles with the spiral symmetry built of several proteins; (iii) large RNA genomes (up to 20 kb); (iv) the presence of a gene for a homolog of HSP70 chaperones; (v) the presence of duplicated genes or gene fragments (e.g., genes for the major and minor coat proteins).

**Genome structure and expression.** The RNA genome of beet yellows virus (BYV) was the first closteroviral genome sequenced. It consists of 14,480 nt and contains the 5′-cap, but lacks the 3′-poly(A) tail [7, 33, 35]. The 5′-terminal portion of BYV genome and genomes of other closteroviruses contain overlapping ORFs 1a and 1b coding for the replication-associated proteins (Fig. 4) [7, 33, 34]. Translation of these genes involves +1 ribosomal frameshifting and results in pp1a and pp1ab. It should be noted that the +1 frameshifting mechanism is rare in the virus world, unlike the –1 frameshifting required for the expression of RNA polymerases of animal retroviruses, nidoviruses, astroviruses, and plant sobemo-like viruses [7, 36], as well as of some eukaryotic genes and transposons [37]. The BYV genes for structural and accessory proteins are located in the 3′-terminal portion of the genome and are expressed via a set of 3′-coterminal sgRNAs [34] (Fig. 4).

The BYV PLP autocatalytically releases the leader protein by cleaving the Gly588/Gly589 bond [7]. Genomes of some other closteroviruses code for duplicated PLP domains, so that two leader proteins are released after the cleavage [8]. The leader protein influences amplification of the BYV RNA, as well as affect the long-distance transmission of the viral infection through the plant conductive tissues [38, 39]. In addition to the cleavage by PLP, pp1a undergoes processing by a yet unknown proteolytic enzyme with the formation of replication-associated proteins of 63 kDa (Mtr) and 100 kDa (HEL) [40].

The major capsid protein (CP) coats ~95% genomic RNA, forming the “body” of the filamentous particle, while the minor capsid protein (CPm) forms a ‘tail’ that includes the 5′-terminal genome portion [41, 42] (Fig. 4). Formation of the BYV particles requires HSP70h and p64. Both proteins (one or several copies) are associated with the mature particles [43-45]. Closteroviral HSP70h has a conserved N-terminal ATPase domain (homologous to the equivalent domains in cell HSP70s) and a variable C-terminal domain [46]. The N-terminal domain of the BYV HSP70h displays the Mg-dependent ATPase activity *in vitro*, but, unlike its cellular orthologs, is unable to interact with unfolded proteins [47]. The BYV HSP70h also interacts with plasmodesmata of infected plant cells and plays a role in the cell-to-cell transmission of the viral infection [48, 49]. Virus transport also depends on p6, p64, CP, and CPm. Knocking out each of the respective genes blocks the cell-to-cell spreading of the BYV infection [50]. The products of the BYV 3′-terminal genes are involved in the long-distance transport of the virus (p20) and suppression of the post-transcriptional gene silencing (p21) [51] (Fig. 4).

**Replication-associated proteins.** Closteroviruses belong to the supergroup of alpha-like viruses that includes (+)RNA viruses of animals (alphaviruses, rubella virus, and hepatitis E virus) and plants (tobacco mosaic virus, brome mosaic virus, and others) (Fig. 1). Despite striking dissimilarities in the biological traits, virion morphology, and genome structure of alpha-like viruses, replicases of these viruses contain conserved Mtr, HEL and POL domains [52, 53]. The Mtr domain has the N^7^-guanine methyltransferase and guanylyltransferase activities and catalyzes the capping of viral RNA. The HEL domain unwinds RNA strands in replication, and the POL domain is responsible for the *de novo* synthesis of complementary RNA strands in a primer-independent fashion [54].

Comparisons of viral (+)RNA reveals a simple rule: the larger the size of genomic RNA, the larger the replicase gene. In other words, replication of large genomes requires more complex RNA replicative complexes [33]. Replicases of alpha-like virus have likely evolved due to the insertion of coding sequences between the fragments encoding the Mtr and HEL domains, whereas the length of the spacer between the HEL and POL domains remained almost unchanged [33]. The replicative complex of closteroviruses is more sophisticated, compared to those of closely related plant viruses, and includes at least five virus-specific proteins (PLP, Mtr, central 1a domain, HEL, and HEL-POL fusion) [34]. Closteroviral pp1a contains no domains equivalent to the nidoviral exonuclease and primase, thus leaving open the question as to whether closteroviruses possess enzymatic activities enhancing the processivity and fidelity of RNA synthesis [51]. Replication of closteroviral genomes possibly follows the mechanism described for other alpha-like viruses: RNA replicase recognizes the 3′-terminal *cis*-signal on the (+)RNA and produces the antigenomic (–)RNA to be further used as a template for the synthesis of progeny genomic and subgenomic RNA strands. No experimental evidence has yet been reported in favor of either of two options of closterovirus transcription, namely, transcription from the subgenomic promoters on the antigenomic (–)RNA template or transcription on the antisubgenomic RNA templates [54]. Obviously, closteroviruses do not employ discontinuous transcription, since their sgRNAs do not have a common 5′-terminal leader sequence, and the subgenomic promoter regions contain no common elements resembling the TRSs of nidoviruses [55-58].

**Cytology of replication.** Closteroviral infection is accompanied by the induction of DMVs (~100 nm in diameter) and MVCs in the cells [59]. These ultrastructures resemble the replication factories of nidoviruses and flaviviruses [60]. The DMVs and MVCs of BYV are produced from the ER membranes. The BYV replicative proteins – PLP, Mtr, and HEL – are associated with the DMVs and MVCs, thus indicating the involvement of these structures in the RNA replication [61, 62]. In search of BYV proteins capable of membrane modification, fragments of the BYV 1a protein fused with the reporter GFP were transiently expressed in *Nicotiana benthamiana* plants [60, 63]. A 198-aa fragment (Mem; Fig. 4) containing the conserved hydrophobic domain with a predicted alpha-helix caused the remodeling of the perinuclear ER membranes and formation of ~2-μm globules. Some globules were mobile and were associated with the actin filaments [63]. It was proposed that remodeling of the ER membranes by the hydrophobic Mem segment of the BYV 1a protein may be one of the steps in the induction of the closteroviral replication-associated ultrastructures in the cells [60, 63].

## EVOLUTION OF LARGE RNA GENOMES

In the course of evolution, animal nidoviruses and plant closteroviruses have exceeded the 12-kb limit of the (+)RNA genome size. Several evolutionary “inventions” have allowed these viruses to solve the problems of packaging and replication of large RNAs. As suggested by Godeny et al*.* [64], the ancestor of extant Nidovirales most likely had an icosahedral nucleocapsid. In the course of subsequent divergence, “small” nidoviruses (13-16 kb RNA) have retained this core structure, whereas “large” nidoviruses (26-41 kb RNA) have acquired the N protein capable of forming helically symmetrical nucleocapsids that allow encapsidation of significantly larger RNAs. The alpha-like plant viruses closely related to *Closteroviridae* have icosahedral (bromoviruses) or rod-like virions (tobamoviruses). The size of RNA that can be packaged into these particles is strictly limited, and it is possible that the evolution of the superflexible closteroviral particles built of several proteins has made it possible to resolve the problem of packaging of larger RNA (up to 19 kb [5].

Mutations introduced by viral RNA polymerases [2] and, possibly, by cellular editing enzymes (e.g., deaminases) [65] are a key factor in the genetic variability of RNA viruses. Another driving force of virus evolution is RNA recombination [66]. Analysis of coronaviral and closteroviral genomes has revealed the obvious traces of recombination events, such as the capture of heterologous sequences and gene duplication. Thus, evolution of “large” nidoviruses has resulted in the acquisition of new enzymatic activities (nucleotidyltransferase, primase, 3′-5′-exonuclease, and endonuclease) [9], as well as of genes for the structural proteins (N protein and outer membrane protein related to the influenza virus hemagglutinin) [67]. Closteroviruses acquired the HSP70 gene (apparently, via recombination with a host mRNA) that has been adapted to perform specific functions in the viral cell-to-cell transmission and particle maturation [33, 51]. In some coronaviruses and closteroviruses, the sequence coding for the leader PLP proteinase has been duplicated. The capsid protein gene in closteroviral genomes has likely been duplicated several times, producing the extant genes for CP, CPm (some family members encode two minor CPs), and p64 (Fig. 4) [34, 51].

The copying of large RNA strands is mediated by more sophisticated replicases, and expansion of the RNA genome is due, for a large part, to the acquisitions in the replication-associated genes [33]. Coronaviruses possess enzymatic activities ensuring an improved fidelity and processivity of RNA replicase (primase and exonuclease). Closteroviral genomes do not encode equivalent enzymes, which might be manifested as a higher rate of point mutations in the citrus tristeza closterovirus (CTV) [68] compared to SARS-CoV and other coronaviruses [65]. Among the Nidovirales, coronaviruses and roniviruses (26-41 kb genome) code for the exonuclease, whereas mesoniviruses and arteriviruses (14-20 kb genome) lack the corresponding domain. Apparently, ~20 kb is a size limit of viral RNA, which requires no additional proofreading activity for its copying.

Comparison of the structure and expression of coronaviral and closteroviral genomes reveals a striking similarity between the two groups (Figs. 1and 4). The genomes of these viruses contain overlapping 5′-terminal replicase genes expressed with via the ribosomal frameshifting to produce large polyproteins. The 1a and 1ab polyproteins are processed by one or two PLPs to release the leader proteins, as well as by the chymotrypsin-like MP (nidoviruses) or protease(s) of unknown origin (closteroviruses), yielding mature proteins with the methyltransferase, helicase, RNA polymerase, and membrane-binding domains. The genes for the accessory and structural proteins of both coronaviruses and closteroviruses are translated using a set of 3′-coterminal sgRNAs (Figs. 1and 4). These similarities do not imply that the two groups of viruses have originated from a common ancestor with similar gene set and expression strategies, as coronaviruses and closteroviruses belong to evolutionary remote lineages [1, 10] (Fig. 1). It is more probable that similar traits of genome organization and expression in coronaviruses and closteroviruses have emerged independently in the course of convergent evolution, along with the RNA genome expansion and acquisition of similar replication-associated functions and gene expression patterns [5, 7, 34, 51].

## Abbreviations

BYV: beet yellows closterovirus
DMV: double-membrane vesicle
MHV: murine hepatitis virus
MVC: multivesicular complex
nsp: nonstructural protein
ORF: open reading frame
pp: polyprotein
RFs: ribosomal frameshifting signal
SARS-CoV: severe acute respiratory syndrome coronavirus
sgRNA: subgenomic RNA
TRS: transcription regulatory signal
